# A new compound heterozygosis for inactivating mutations in the glucokinase gene as cause of permanent neonatal diabetes mellitus (PNDM) in double-first cousins

**DOI:** 10.1186/s13098-015-0101-9

**Published:** 2015-11-18

**Authors:** Adriana Mangue Esquiaveto-Aun, Maricilda Palandi De Mello, Maria Fernanda Vanti Macedo Paulino, Walter José Minicucci, Gil Guerra-Júnior, Sofia Helena Valente De Lemos-Marini

**Affiliations:** Department of Pediatrics, School of Medical Sciences (FCM), State University of Campinas (UNICAMP), Campinas, SP Brazil; Center of Molecular Biology and Genetic Engineering (CBMEG), UNICAMP, Campinas, Brazil; Center for Investigation in Pediatrics (CIPED), FCM, UNICAMP, Campinas, Brazil; Division of Endocrinology, Department of Clinical Medicine, FCM, UNICAMP, Campinas, Brazil

**Keywords:** Permanent neonatal diabetes mellitus, PNDM, MODY, Glucokinase, GCK

## Abstract

**Background:**

Permanent neonatal diabetes mellitus (PNDM) is a rare disorder, characterized by uncontrolled hyperglycemia diagnosed during the first 6 months of life. In general, PNDM has a genetic origin and most frequently it results from heterozygous mutations in *KCNJ11*, *INS* and *ABCC8* genes. Homozygous or compound heterozygous inactivating mutations in *GCK* gene as cause of PNDM are rare. In contrast, heterozygosis for *GCK* inactivating mutations is frequent and results in the maturity-onset diabetes of young (MODY), manifested by a mild fasting hyperglycemia usually detected later in life. Therefore, as an autosomal recessive disorder, GCK-PNDM should be considered in families with history of glucose intolerance or MODY in first relatives, especially when consanguinity is suspected.

**Results:**

Here we describe two patients born from non-consanguineous parents within a family. They presented low birth weight with persistent hyperglycemia during the first month of life. Molecular analyses for *KCNJ11,INS, ABCC8* did not show any mutation. *GCK* gene sequencing, however, revealed that both patients were compound heterozygous for two missense combined in a novel GCK-PNDM genotype. The p.Asn254His and p.Arg447Gly mutations had been inherited from their mothers and fathers, respectively, as their mothers are sisters and their fathers are brothers. Parents had been later diagnosed as having GCK-MODY.

**Conclusions:**

Mutations’ in silico analysis was carried out to elucidate the role of the amino acid changes on the enzyme structure. Both p.Asn254His and p.Arg447Gly mutations appeared to be quite damaging. This is the first report of GCK-PNDM in a Brazilian family.

## Background

Permanent neonatal diabetes mellitus (PNDM; OMIM #606176) is described as a severe monogenic form of diabetes. It is diagnosed during the first 6 months of life and requires lifelong treatment with insulin and/or high doses of sulfonylurea [1, 2]. In Europeans, the estimated incidence rate is about one case per 200,000–250,000 live births [3–5]. It is a rare condition associated with defects in genes that play major roles in the development, survival and function of pancreatic beta cells. Mutations in about 12 different genes have been identified as responsible for PNDM [2].

Most frequently, heterozygous mutations in the *KCNJ11* (OMIM 600937), *INS* (OMIM 176730) and *ABCC8* (OMIM 600509) genes are associated with PNDM [4]. Despite significant progress in the elucidation of the molecular basis of PNDM during last decade, up to 40 % of patients remain with unknown etiology [6–8].

Homozygous or compound heterozygous mutations in the *GCK* gene (OMIM *138079) are considered a rare cause of PNDM with an autosomal recessive inheritance [9]. Heterozygous inactivating mutations in *GCK*, however, cause an autosomal dominant subtype of maturity onset diabetes of young (MODY), subtype glucokinase (GCK-MODY, previously termed MODY 2; OMIM #125851). GCK-MODY is characterized by mild fasting hyperglycemia, usually diagnosed later in life [10]. The *GCK*-PNDM phenotype, however, is more severe with hyperglycemia manifesting after birth [11–13]. Conversely, activating heterozygous *GCK* mutations have been reported as cause of an opposite phenotype, characterized by inappropriate over secretion of insulin despite hypoglycemia, called hyperinsulinemic hypoglycemia (HH) [14], with a growing number of mutations being reported ever since [15]. More than 600 *GCK* mutations have been described in families with PNDM, MODY or HH, all of them distributed throughout the gene, with no mutation “hot spots” [15].

*GCK* gene is located in the 7p15.3–p15.1 chromosomal region and comprises 12 exons and 11 introns which span ~45,168 bp. The encoded protein has 465 amino acids with a molecular weight of 52,191 Da and it is expressed mainly in the pancreas, liver and brain [16]. The presence of tissue-specific promoters allows differential regulation and transcription of different transcripts giving rise to three different-sized exon 1 (a, b, and c). Exon 1b and 1c are expressed in the liver while exon 1a is expressed in the pancreatic beta cells [17].

Glucokinase (GCK; EC 2.7.1.1), a hexokinase IV or D, is a cytoplasmatic enzyme that catalyses the conversion of glucose to glucose-6-phosphate in the first reaction of the glycolytic pathway. It differs from the other members of hexoquinase family of enzymes (hexokinases I, II and III or hexokinases A, B, and C) due to its peculiar kinetic properties. Known as the glucose sensor in liver and pancreatic beta cells, GCK is a key regulatory monomeric enzyme that displays low affinity for glucose and a sigmoidal saturation curve for its substrate. In the liver, the glucose phosphorylation by GCK promotes glycogen synthesis, while in pancreatic beta cells it results in the release of insulin. Both effects, in turn, reduce plasma glucose levels [18, 19]. The rate of glucose phosphorylation in pancreatic beta cells is directly related to the concentration of glucose over a range of physiological glucose concentrations (4–15 mmol/L) and it is a rate-limiting step in the insulin secretion [18].

Pancreatic beta cells are able to regulate the rate of glucose metabolism in response to extracellular glucose concentration. In order to maintain glucose metabolism there must be a rapid equilibration across the plasma membrane and GCK glucose phosphorylation [20]. Given its central role in the regulation of insulin release, it is understandable that inactivating or activating mutations in the *GCK* gene may cause either hyper or hypoglycemia, respectively [21].

Here we describe a non-consanguineous Brazilian family with two cases of PNDM diagnosed during first months of life, whose parents showed mild fasting hyperglycaemia (GCK-MODY) during investigation. *GCK* gene sequencing revealed two missense mutations in both index cases: p.Asn254His was inherited from their mothers, who are sisters; and p.Arg447Gly, also identified in one sibling was inherited from their fathers, who are brothers as well.

## Case reports

### Subject 1

**Fig. 1:**
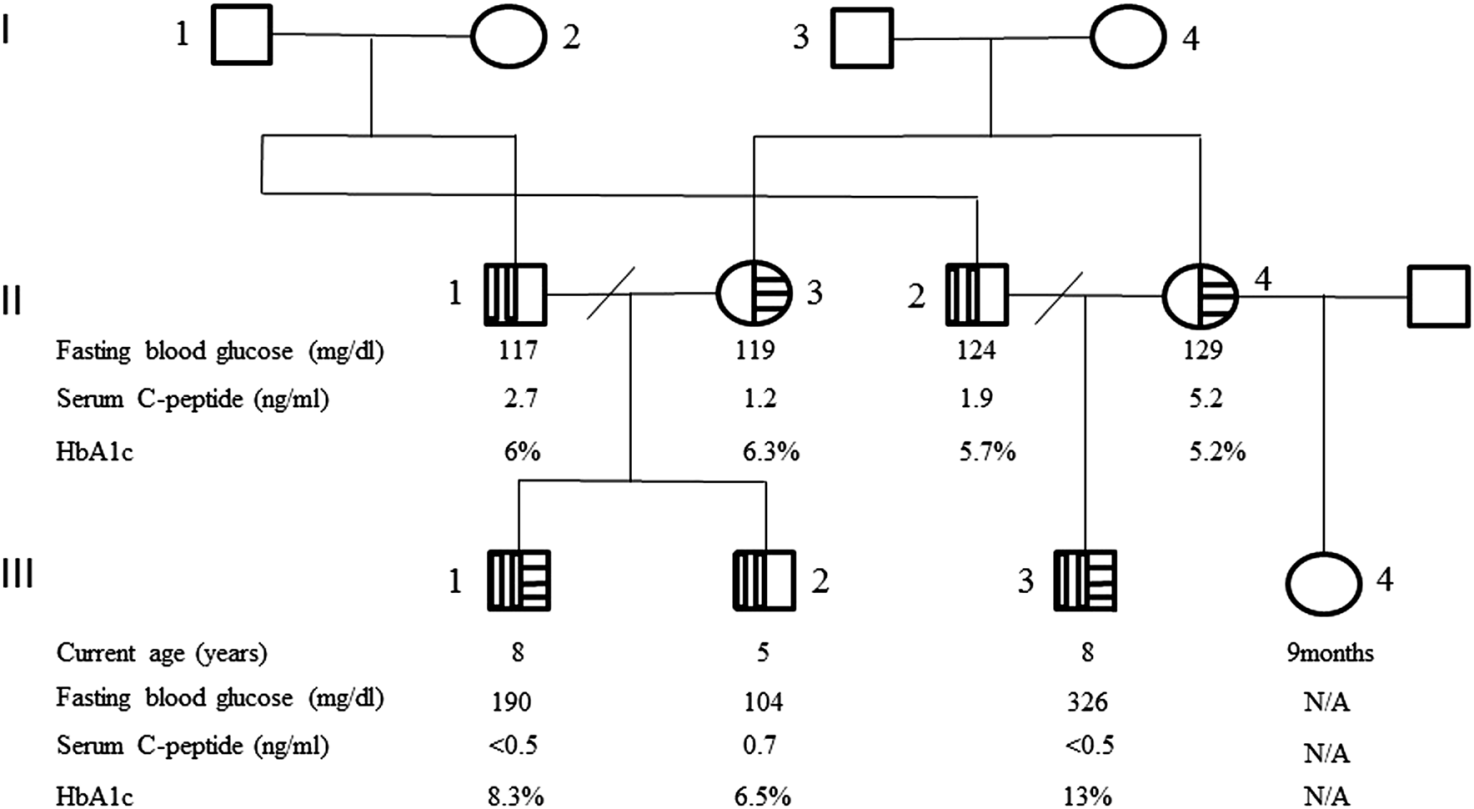
Pedigree showing inheritance of *GCK* mutations. *Squares with vertical stripes* denote males heterozygous for p.Arg447Gly, with GCK-MODY. *Circles with horizontal stripes* indicate females heterozygous for p.Asn254His, with GCK-MODY. Probands are indicated by *arrows*. N/A means data not available. Normal range for laboratorial tests: fasting blood glucose 75–99 mg/dl; serum C-peptide 0.8–4 ng/ml; haemoglobin A1c 3.9–6.1 % (19–43 mmol/mol)

The male proband (Subject III-1, Fig. 1) was referred for evaluation at 2 months of age because severe hyperglycemia initiated during the 31 days of life [venous glucose level of 700 mg/dL (38.8 mmol/L)]. He was born at term by vaginal delivery with a birth weight of 1750 g and 42 cm of length, consistent with intrauterine growth restriction (IUGR), after a nonsupervisioned pregnancy. The mother was drug addict and HIV seropositive. Insulin treatment was initiated at diagnosis with a NPH regimen and regular insulin injections twice a day (0.32 U/kg/day). Further investigation at 8 months of age indicated undetectable serum C-peptide level [<0.5 ng/mL (normal range 0.8–4 ng/mL)] and negative diabetes-associated autoantibodies (i.e., against insulin, islet cell or glutamic acid decarboxylase). He is now 10 years old, under a basal–bolus insulin therapy, with poor glycemic control due to a bad diet compliance and adverse social condition. The high HbA1c level [8.3 % (67 mmol/mol)−normal range 3.9–6.1 %] comprove poor glycemic control. Mental-motor development is normal and his weight and height is within 3rd and 10th percentiles, respectively. During investigation, his mother, father and his younger brother presented impaired fasting glycaemia (Fig. 1).

### Subject 2

The male proband (Subject III-3, Fig. 1), 1 month younger cousin of subject 1, was first seen at 1 month of age because hyperglycemia diagnosed at 26 days of life. He was born at term by caesarean section due to intrauterine growth restriction (birth weight, 1550 g; length, 40.5 cm). Pregnancy underwent without medical control and his mother was also drug addict. At diagnosis he presented a high glucose level [1400 mg/dL (77.7 mmol/L)], respiratory distress, hyperthermia and dehydration (tachycardia and hypotension). After fluid replacement therapy and metabolic support, insulin therapy (with NPH and regular insulins—0.4 U/kg/day) was started to control hyperglycemia. On further investigation, the serum C-peptide level was likewise undetectable (<0.5 ng/mL) and the diabetes-associated autoantibodies were negative. He is now 10 years old and is receiving a basal-bolus insulin therapy. Achieving good glycemic control was proving difficult due to the same poor diet compliance and social problems, with an elevated haemoglobin A1c [HbA1c—13 % (119 mmol/mol)]. He has a good neuro-motor development and an appropriate growth (weight and height at 25th percentile). Laboratory tests revealed mild fasting hyperglycemia in both mother and father (Fig. 1). His mother had a history of gestational diabetes in her last pregnancy, from a new relationship.

## Methods

Genomic DNAs from patients, parents and sibling were purified from peripheral leukocytes by proteinase K lysis, phenol/chloroform extraction, and ethanol precipitation, according to standard techniques. Sequencing of genes such as *KCNJ11*, *INS* and *ABCC8* that encode, respectively, Kir6.2, the inward rectifier subunit of the ATP-sensitive potassium channel of the beta cells, preproinsulin and SUR1, the regulatory subunit of the ATP-sensitive potassium channel of the beta cells had been performed as described elsewhere [22–24]. The complete coding region for isoform 1 of *GCK* that is expressed in pancreas was sequenced. Fragments containing the ten exons, and the 5′ and 3′ untranslated flanking regions were amplified by polymerase chain reaction (PCR) using specific primers designed based on the reference gene sequence (ENSG00000106633, http://www.ensembl.org). Independent PCR fragments were purified in 1 % agarose gel electrophoresis with the wizard SV gel and PCR clean-up system (Promega, Madison, WI, USA), and both sense and antisense strands were sequenced using the BigDye Terminator v3.1 Cycle sequencing kit (Life Technologies, Grand Island, NY, USA) with the same primers used for PCR reactions. The Chromas Lite 2.0 (Technelysium Pty Ltd) and CLC Sequence Viewer v.6.8.1 free software (CLC bio) were used to analyze and compare sequences with the reference *GCK* sequence. Structural analyses were performed using PDB ID: 4DCH—chain A as template [25]. The native and mutant models were obtained by SWISS MODEL web-served program. Internal contacts were evaluated by STING Millennium (http://www.cbi.cnptia.embrapa.br) and visualized by PyMol^®^. Two predictive methods to evaluate the effect of the amino acid substitutions were used: polymorphism phenotyping (PolyPhen) that gives scores ranging from 0 (neutral) to a positive (damaging) number and sorting intolerant from tolerant (SIFT) whose scores range from 0 (damaging) to 1 (neutral) [26].

## Results

**Fig. 2:**
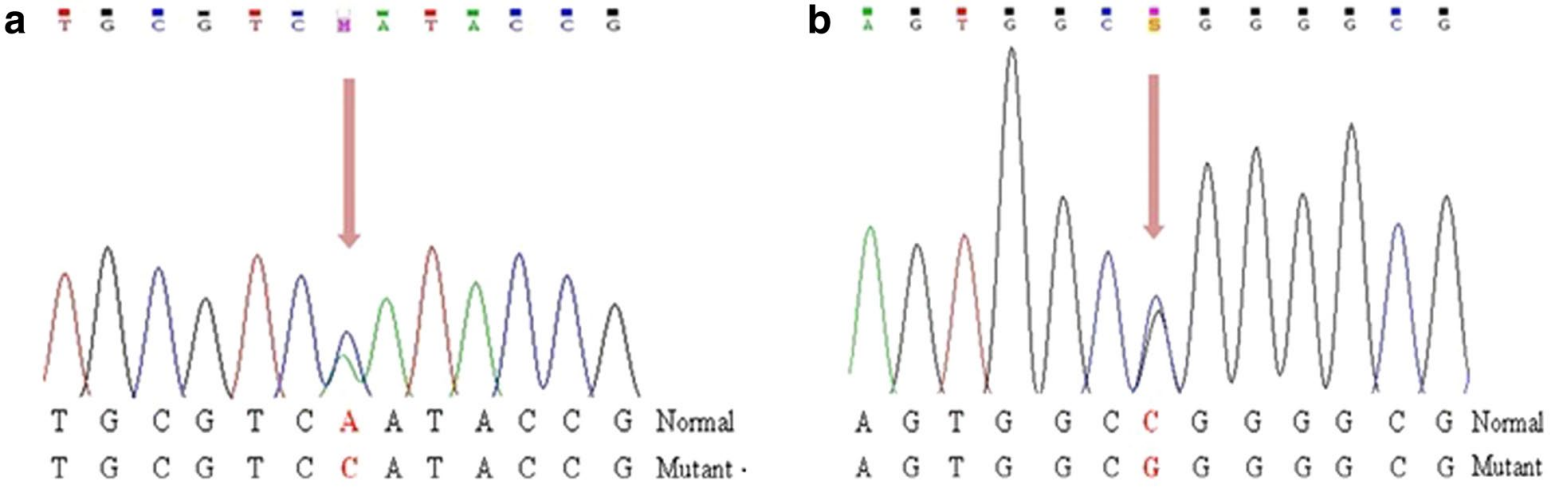
**a** Electropherogram showing part of *GCK* exon 7 sequence where the c.760A>C heterozygous transversion leading to p.Asn254His mutation occurred. **b** Electropherogram showing part of *GCK* exon 10 sequence where the c.1340C>G heterozygous transition leading to p.Arg447Gly mutation occurred

DNA sequence analyses of *KCNJ11, INS* and *ABCC8* genes did not show any mutation. However, *GCK* gene sequencing revealed two novel transversions: A>C within exon 3 and C>G within exon 10 in both probands (Fig. 2). The first was also identified in their mothers who are sisters; while the second was identified in their fathers, who are brothers, and in the brother of subject 1 (Subject III-2, Fig. 1). The nucleotide change c.760A>C is predicted to cause the substitution of an asparagine by a histidine at the residue 254 (p.Asn254His) while the nucleotide change c.1340C>G is predicted to cause the substitution of an arginine by a glycine at the amino acid residue 447 (p.Arg447Gly).

The crystal structure of human GCK was solved in 2004 and revealed that GCK has a large and a small domain forming a deep cleft where glucose binds [19]. The binding-site within this cleft involves residues Glu256 and Glu290 in the large domain, Thr168 and Lys169 in the small domain and Asn204 and Asp205 in the connecting region I. Induced by glucose binding, GCK undergoes a global conformational change, with the large and small domains getting physically closer and the structure assumes a closed active conformation [19].

**Fig. 3:**
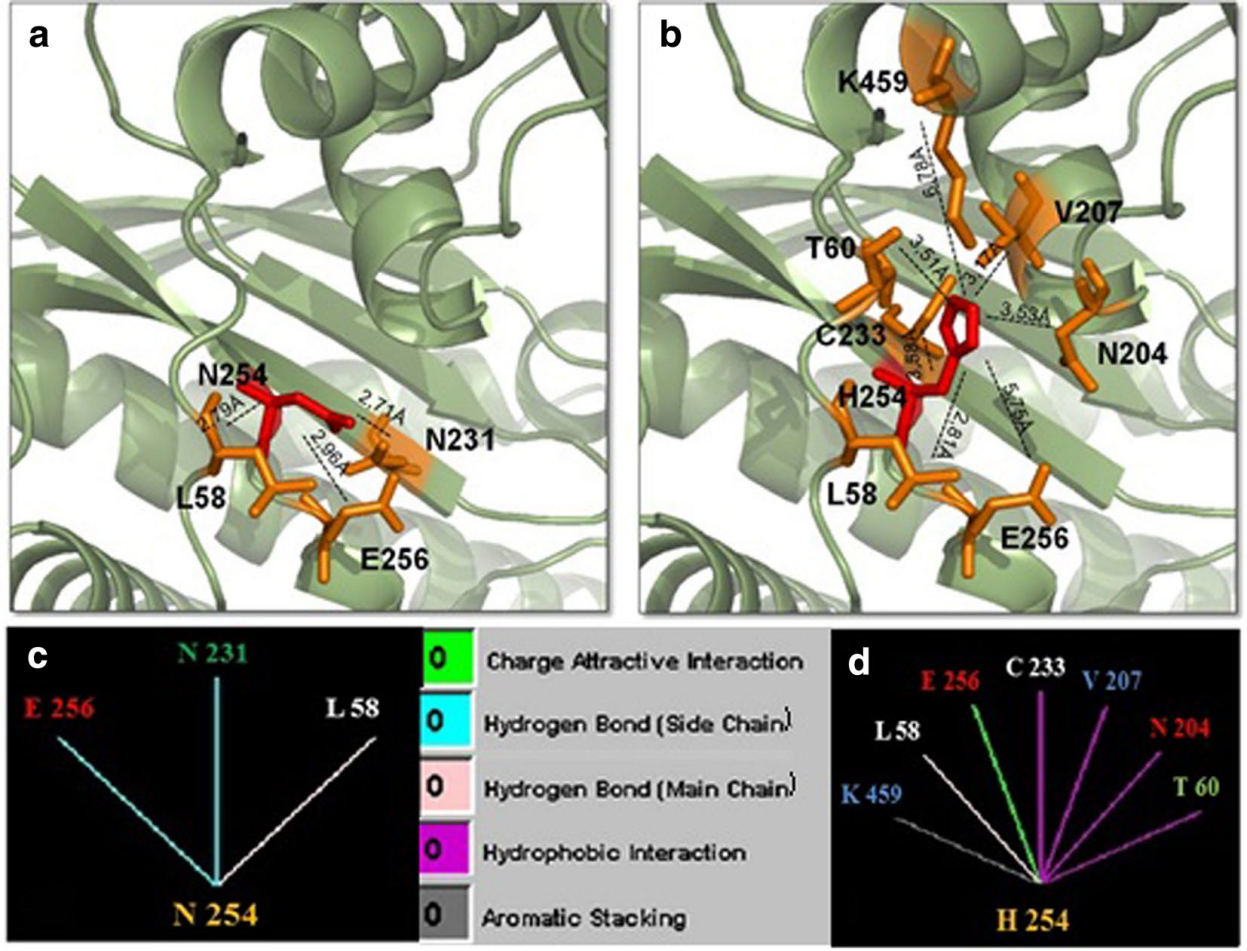
Comparison of normal and mutant glucokinase models at residue 254. **a**, **b** Pymol structures for normal asparigine and mutant histidine, respectively. Each residue is denoted in *red* and surrounding residues in *orange*. *Dashed lines* represent the distance in Ångström (Å) for internal contacts. **c**, **d** Graphic results for residue interactions obtained in the STING Millennium analysis. **a**, **c** The native Asn254 forms hydrogen bond interactions with E256, N231 and L58. **b**, **d** The mutant His254 suppresses the interaction with N231, maintains the contact with L58, introduces four additional hydrophobic interactions with C233, V207, N204 and T60, creates a new aromatic stacking with K459 and changes the hydrogen bond wiht E256 to a charge attractive interaction. Between **c** and **d,** *color legend* for internal interactions provided by BlueStar STING software

Residue 254, where the p.Ans254His occurred, is located in the large domain within the GCK cleft region, two residues away from Glu256 that is critical for glucose binding. Structural analyses demonstrated that the native Asn254 makes internal hydrogen bond interactions with Glu256, Asn231 and Leu58 (Fig. 3a, c). The mutant His254 seems to disturb drastically the nearby structure. Since it is a bulkier amino acid the distance between residue 254 and residues Leu58 and Glu256 increases and the interaction with Asn231 is lost. Additionally, it established new interactions with residues Thr60, Lys459, Cys233, Val207 and Asn204, which is also part of the active phosphorylation glucose binding-site (Fig. 3b, d).

**Fig. 4:**
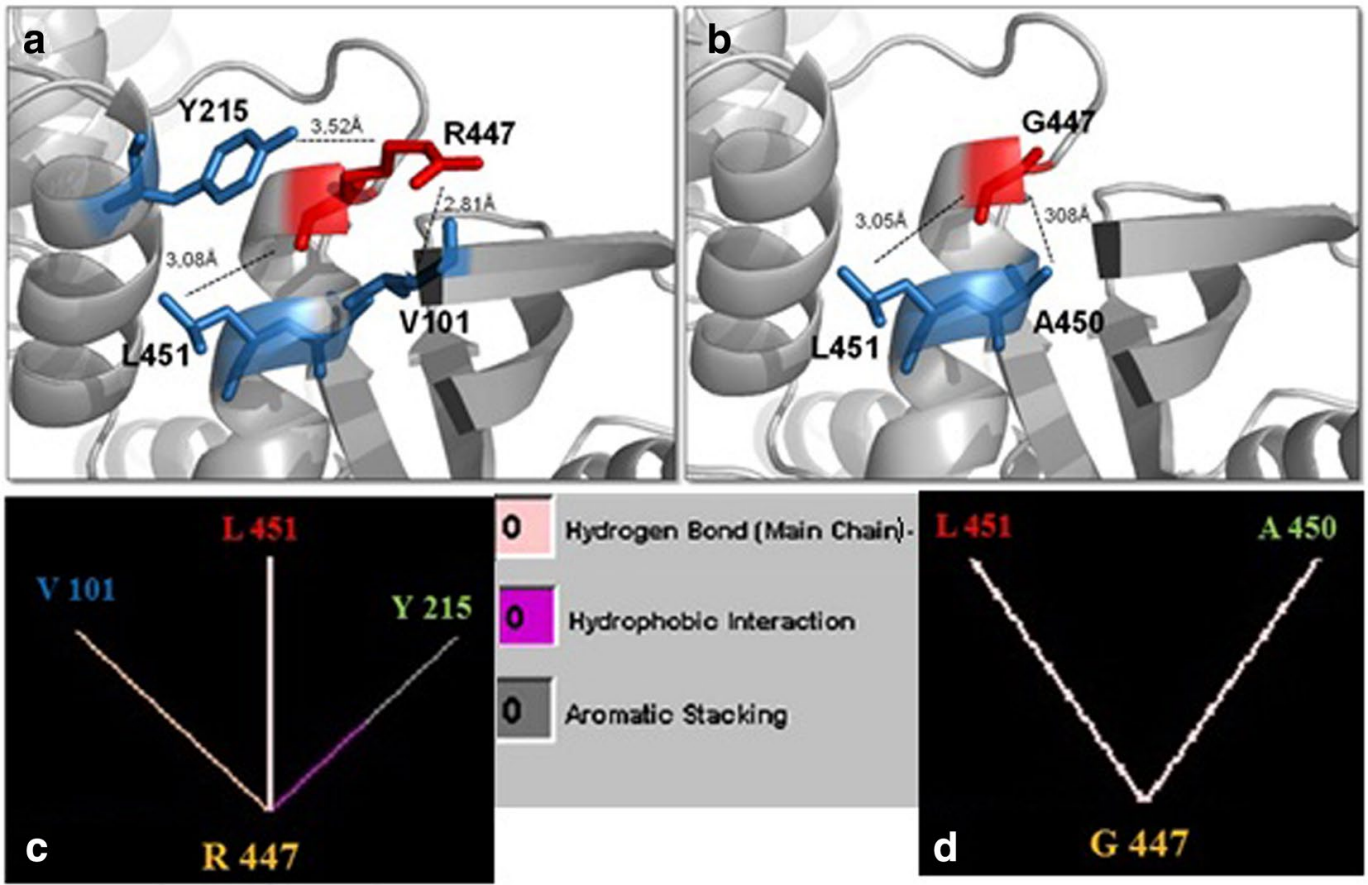
Comparison of normal and mutant glucokinase models at residue 447. **a**, **b** Pymol structures for normal arginine and mutant glycine, respectively. Each residue is denoted in *red* and surrounding residues in *blue*. *Dashed lines* represent the distance in Ångström (Å) for internal contacts. **c**, **d** Graphic results for residue interactions obtained in the STING Millennium analysis. **a**, **c** The native Arg447 makes two hydrogen bonds with Val101 and with Leu451, and interacts with Tyr215 through aromatic stacking and hydrophobic interactions. **b**, **d** The mutant Gly447 looses both Val101 and Tyr215 interactions, creates a new hydrogen bond with Ala450, and maintains the hydrogen bond with Leu451. Between **c** and **d**, *color legend* for internal interactions provided by BlueStar STING software

Residue 447 is located at the edge of the α-13 helix in the C-terminal region of glucokinase that is part of the small domain [27]. The native Arg447 makes two hydrogen bonds with Val101 and with Leu451, and interacts with Tyr215 throught aromatic stacking and hydrophobic interactions (Fig. 4a, c). Conversely, the mutant Gly447 loses both Val101 and Tyr215 interactions, creates a new hydrogen bond with Ala450, whether maintains the hydrogen bond with Leu451 (Fig. 4b, d).

To estimate functional damage caused by each mutation in silico analysis with different algorithms were performed. For p.Asn254His mutation scores of 0.986 and 0 were obtained in PolyPhen and SIFT analyses, respectively. Similar results were obtained for p.Arg447Gly mutation whose scores were 0.998 and 0 for PolyPhen and SIFT, respectively.

## Discussion

We describe here two first-cousins with PNDM, diagnosed at the first month of life due to severe hyperglycemia. They were compound heterozygous for missense mutations in *GCK* gene. To our knowledge, this report contains the first description on GCK mutations in Brazilian patients with non-consanguineous parents. Other cases of GCK-PNDM have been described in consanguineous Arabian and European GCK-MODY families and, to date, a total of nine GCK-PNDM related mutations have been reported [11–13, 28–31]. European cases of PNDM have been reviewed and verified that complete deficiency of glucokinase is not a common cause of PDMN [9, 32].

Initially, *KCNJ11, INS* and *ABCC8* gene screenings were performed to investigate the genetic origin of PNDM in the present patients, since mutations in those genes are most frequently found as cause of the disease considering that GCK-PNDM is a rarer condition, especially when parents are not consanguineous. Since no mutations in those genes had been identified, we decided to evaluate the glucose fasting level of the parents, who were asymptomatic and unwilling to be investigated at first. Unexpectedly, they showed a mild fasting hyperglycemia when we so decided to perform *GCK* sequencing. The p.Asn254His and p.Arg447Gly identified in both patients have been inherited from their mothers, who were sisters, and their fathers, who were brothers, respectively. The p.Arg447Gly was also identified in one sibling.

The asparagine in residue 254 is within the large domain of GCK [19]. In the wild-type model, this residue interacts with Glu256 maintaining a distance of 2.96 Å. The residue Glu256 is critical for GCK activity since it is part of glucose binding site in the cleft interdomain [19]. According to structural analyses, the substitution of an asparagine by a histidine at residue 254 modifies the interaction and the distance (5.75 Å) with Glu256. The disturbance in the glucose binding site conformation certainly reflects in all the surroundings. The hydrogen bound with Leu58 did not modify significantly the distance between the residues in the wild-type and mutant models (2.79 and 2.81 Å, respectively). In contrast, the hydrogen bound with Asn231 was suppressed and five new internal interactions can be observed in the mutant model, as histidine is a bulky amino acid compared to the original asparagine. A novel aromatic interaction with Lys459 and four hydrophobic bonds with Cys233, Val207, Thr60 and Asn204 can be observed in the mutant. Additionally, the Asn204 residue is located on connecting region I that is also part of the glucose binding-site in GCK. Upon solving the crystal structure of human GCK by Kamata and colleagues [19] it became well understood that this enzyme assumes three structural conformations: closed, open (both actives) and super-open form (inactive). When glucose binds to the super-open form, GCK undergoes to a large conformational change to the open form. In the presence of ATP, GCK acquire the closed conformation, and the phosphorylation of glucose to glucose-6-phosphate is carried out. After the reaction is complete, GCK returns to its open form in order to release glucose-6-phosphate and ADP. If glucose binds to the open form during this period, GCK re-enters the catalytic cycle. If this does not happen, GCK returns to the inactive super-open form. Therefore, the flexibility of the enzyme is critical for its activity. Here we postulate that p.Asn254His may compromise the GCK phosphorylating potential by affecting its binding-site, since the mutation modifies several interactions with surrounding residues in this critical region. Moreover, when other internal contacts that restrict the enzymatic flexibility are created, a consequent damage affecting its activity may occur.

The arginine at codon 447 is described as a component of the α-13 helix of human GCK that extends from residue Glu443 to Ala460 [27]. The α-13 and α-5 helices, within the small domain, play a critical role in the conformational change that occurs between the active and inactive forms of the enzyme [19]. In the closed, active form of GCK, the α-13 helix is included in the small domain but in the super-open form, the inactive conformation, the α-13 helix is released from the small domain because of the loose structure of connecting region I, in order to facilitate its large rotation. Given that the α-13 helix is crucial for the conformational changes observed during the enzymatic activation, sequence divergences in this region is supposed to disturb its function. The p.Arg447Gly led to a new internal contact, a hydrogen bond with Ala450. Although the hydrogen bond with Leu451 residue was maintained, two other interactions were suppressed. Mutations are often observed in this region as activating mutations related to HH [33]. However, two heterozygous inactivating mutations in residue 447 (p.Arg447Gln and p.Arg447del29) have already been described as cause of GCK-MODY [34]. Recently, Capuano et al. [35] described the frameshift mutation p.Arg447Glyfs and Shammas et al. [36] reported the missense mutation p. Arg447Pro, as cause of GCK-MODY phenotype as well. The missense mutation p.Arg447Gly is been reported here for the first time, as cause of GCK-MODY in three heterozygous members of the family and also as cause of GCK-PNDM in association with p.Asn254His mutation, described here for the first time as well. The identification of *GCK* mutations in patients with PNDM provides a firm diagnosis of the subtype and, as any inherited disorder, brings implications for diagnosis and treatment of other members in a family. Recently, some authors described an improvement in the glycaemic control in response to oral sulphonylurea therapy in association with insulin in patients with GCK-PNDM, with an increase in both basal and stimulate insulin secretion [13, 30]. Besides, efforts to replace treatment with insulin by sulfonylurea (as in cases of neonatal diabetes caused by mutations in *KCNJ11* and *ABCC8* genes) were not successful since the improvement in glycemic control was partial and insulin injections could not be stopped.

The discovery of inactivating and activating *GCK* mutations and their functional implications has led to important insights into the biochemical GCK activation and regulation [15] and its central role in glucose homeostasis. Specially, mutations in α-13 helix as it is the case for p.Arg447Gly encourage the development of drugs that specifically target this GCK region and also highlights some key interactions between α-13 and 5 that may be suitable for drug-induced modulation [36]. Many efforts in this area have been done to identify novel pharmacological agents that could activate glucokinase by a similar mechanism to that observed for mutations leading to GCK-HH. The discovery of several classes of small molecular glucokinase activators (GKAs) that showed to enhance insulin release and reduce hepatic glucose production in mouse model, has been seen as a potential target for antidiabetic therapy [37]. However, recent clinical trials revealed that GKAs lose their efficacy over time and their use is associated with high incidence of hypoglycemia as well as dyslipidemia and hepatic steatosis [38].

## References

[1] Edghill EL, Dix RJ, Flanagan SE, Bingley PJ, Hattersley AT, Ellard S (2006). HLA genotyping supports a nonautoimmune etiology in patients diagnosed with diabetes under the age of 6 months. Diabetes.

[2] Rubio-Cabezas O, Klupa T, Malecki MT, Consortium C (2011). Permanent neonatal diabetes mellitus—the importance of diabetes differential diagnosis in neonates and infants. Eur J Clin Invest.

[3] Stanik J, Gasperikova D, Paskova M, Barak L, Javorkova J, Jancova E (2007). Prevalence of permanent neonatal diabetes in Slovakia and successful replacement of insulin with sulfonylurea therapy in KCNJ11 and ABCC8 mutation carriers. J Clin Endocrinol Metab.

[4] Slingerland AS, Shields BM, Flanagan SE, Bruining GJ, Noordam K, Gach A (2009). Referral rates for diagnostic testing support an incidence of permanent neonatal diabetes in three European countries of at least 1 in 260,000 live births. Diabetologia.

[5] Russo L, Iafusco D, Brescianini S, Nocerino V, Bizzarri C, Toni S (2011). Permanent diabetes during the first year of life: multiple gene screening in 54 patients. Diabetologia.

[6] Naylor RN, Greeley SAW, Bell GI, Philipson LH (2011). Genetics and pathophysiology of neonatal diabetes mellitus. J Diab Invest.

[7] Kanakatti Shankar R, Pihoker C, Dolan LM, Standiford D, Badaru A, Dabelea D (2013). Permanent neonatal diabetes mellitus: prevalence and genetic diagnosis in the SEARCH for diabetes in youth study. Pediatr Diab.

[8] Oriola J, Moreno F, Gutiérrez-Nogués A, León S, García-Herrero CM, Vincent O (2015). Lack of glibenclamide response in a case of permanent neonatal diabetes caused by incomplete inactivation of glucokinase. JIMD Rep.

[9] Gloyn AL, Ellard S, Shield JP, Temple IK, Mackay DJ, Polak M (2002). Complete glucokinase deficiency is not a common cause of permanent neonatal diabetes. Diabetologia.

[10] Froguel P, Zouali H, Vionnet N, Velho G, Vaxillaire M, Sun F (1993). Familial hyperglycemia due to mutations in glucokinase. Definition of a subtype of diabetes mellitus. N Engl J Med.

[11] Njølstad PR, Søvik O, Cuesta-Muñoz A, Bjørkhaug L, Massa O, Barbetti F (2001). Neonatal diabetes mellitus due to complete glucokinase deficiency. N Engl J Med.

[12] Njølstad PR, Sagen JV, Bjørkhaug L, Odili S, Shehadeh N, Bakry D (2003). Permanent neonatal diabetes caused by glucokinase deficiency: inborn error of the glucose-insulin signaling pathway. Diabetes.

[13] Turkkahraman D, Bircan I, Tribble ND, Akçurin S, Ellard S, Gloyn AL (2008). Permanent neonatal diabetes mellitus caused by a novel homozygous (T168A) glucokinase (GCK) mutation: initial response to oral sulphonylurea therapy. J Pediatr.

[14] Glaser B, Kesavan P, Heyman M, Davis E, Cuesta A, Buchs A (1998). Familial hyperinsulinism caused by an activating glucokinase mutation. N Engl J Med.

[15] Osbak KK, Colclough K, Saint-Martin C, Beer NL, Bellanné-Chantelot C, Ellard S (2009). Update on mutations in glucokinase (GCK), which cause maturity-onset diabetes of the young, permanent neonatal diabetes, and hyperinsulinemic hypoglycemia. Hum Mutat.

[16] Iynedjian PB (1993). Mammalian glucokinase and its gene. Biochem J.

[17] Magnuson MA (1990). Glucokinase gene structure. Functional implications of molecular genetic studies. Diabetes.

[18] Matschinsky FM (2002). Regulation of pancreatic beta-cell glucokinase: from basics to therapeutics. Diabetes.

[19] Kamata K, Mitsuya M, Nishimura T, Eiki J, Nagata Y (2004). Structural basis for allosteric regulation of the monomeric allosteric enzyme human glucokinase. Structure.

[20] Hussain K (2010). Mutations in pancreatic β-cell glucokinase as a cause of hyperinsulinaemic hypoglycaemia and neonatal diabetes mellitus. Rev Endocr Metab Disord..

[21] Gloyn AL, Noordam K, Willemsen MA, Ellard S, Lam WW, Campbell IW (2003). Insights into the biochemical and genetic basis of glucokinase activation from naturally occurring hypoglycemia mutations. Diabetes.

[22] Gloyn AL, Pearson ER, Antcliff JF, Proks P, Bruining GJ, Slingerland AS (2004). Activating mutations in the gene encoding the ATP-sensitive potassium-channel subunit Kir6.2 and permanent neonatal diabetes. N Engl J Med.

[23] Støy J, Edghill EL, Flanagan SE, Ye H, Paz VP, Pluzhnikov A (2007). Insulin gene mutations as a cause of permanent neonatal diabetes. Proc Natl Acad Sci U S A..

[24] Babenko AP, Polak M, Cavé H, Busiah K, Czernichow P, Scharfmann R (2006). Activating mutations in the ABCC8 gene in neonatal diabetes mellitus. N Engl J Med.

[25] Liu S, Ammirati MJ, Song X, Knafels JD, Zhang J, Greasley SE (2012). Insights into mechanism of glucokinase activation: observation of multiple distinct protein conformations. J Biol Chem.

[26] Ng PC, Henikoff S (2006). Predicting the effects of amino acid substitutions on protein function. Annu Rev Genomics Hum Genet.

[27] Pedelini L, Garcia-Gimeno MA, Marina A, Gomez-Zumaquero JM, Rodriguez-Bada P, López-Enriquez S (2005). Structure-function analysis of the alpha5 and the alpha13 helices of human glucokinase: description of two novel activating mutations. Protein Sci.

[28] Porter JR, Shaw NJ, Barrett TG, Hattersley AT, Ellard S, Gloyn AL (2005). Permanent neonatal diabetes in an Asian infant. J Pediatr.

[29] Rubio-Cabezas O, Díaz González F, Aragonés A, Argente J, Campos-Barros A (2008). Permanent neonatal diabetes caused by a homozygous nonsense mutation in the glucokinase gene. Pediatr Diab.

[30] Bennett K, James C, Mutair A, Al-Shaikh H, Sinani A, Hussain K (2011). Four novel cases of permanent neonatal diabetes mellitus caused by homozygous mutations in the glucokinase gene. Pediatr Diab.

[31] Wajda-Cuszlag M, Witkowski D, Piontek E, Wysocka-Mincewicz M, Borowiec M, Młynarski W (2012). Glucokinase gene mutation as a causative factor of permanent neonatal diabetes mellitus. Pediatr Endocrinol Diab Metab.

[32] Vaxillaire M, Samson C, Cavé H, Metz C, Froguel P, Polak M (2002). Glucokinase gene mutations are not a common cause of permanent neonatal diabetes in France. Diabetologia.

[33] Gloyn AL (2003). Glucokinase (GCK) mutations in hyper- and hypoglycemia: maturity-onset diabetes of the young, permanent neonatal diabetes, and hyperinsulinemia of infancy. Hum Mutat.

[34] Thomson KL, Gloyn AL, Colclough K, Batten M, Allen LI, Beards F (2003). Identification of 21 novel glucokinase (GCK) mutations in UK and European Caucasians with maturity-onset diabetes of the young (MODY). Hum Mutat.

[35] Capuano M, Garcia-Herrero CM, Tinto N, Carluccio C, Capobianco V, Coto I (2012). Glucokinase (GCK) mutations and their characterization in MODY2 children of southern Italy. PLoS ONE.

[36] Shammas C, Neocleous V, Phelan MM, Lian LY, Skordis N, Phylactou LA (2013). A report of 2 new cases of MODY2 and review of the literature: implications in the search for type 2 diabetes drugs. Metabolism.

[37] Doliba NM, Fenner D, Zelent B, Bass J, Sarabu R, Matschinsky FM (2012). Repair of diverse diabetic defects of β-cells in man and mouse by pharmacological glucokinase activation. Diab Obes Metab.

[38] Matschinsky FM (2013). GKAs for diabetes therapy: why no clinically useful drug after two decades of trying?. Trends Pharmacol Sci.

